# Factors associated with patients’ experience of accessibility to general practice: results from a national survey in Norway

**DOI:** 10.1186/s12913-024-11460-8

**Published:** 2024-08-30

**Authors:** Elma Jelin, Oyvind Bjertnaes, Rebecka Maria Norman

**Affiliations:** Norwegian Institute of Public Health, Skoyen, PO Box 222, Oslo, 0213 Norway

**Keywords:** General practitioner, Accessibility of healthcare services, Access to health care, Continuity, Patient experience, Patient-reported experience measures

## Abstract

**Background:**

This study aimed to explore the influence of patient-, general practitioners (GP)-, and GP practice-level predictor variables on patient-experienced accessibility to GPs and GP practices. Additionally, we aimed to enhance our understanding of patient-experienced accessibility by analysing the free-text comments from patients who reported lowest accessibility scores to GPs and GP practices.

**Methods:**

We performed a secondary analysis of data from a 2021-2022 national Norwegian survey on patient experiences with their GP and GP practice. We identified seven accessibility-related items including experience and acceptance of regular waiting time and for urgent appointments, time spent with the GP, waiting time in the wating-room, and getting in touch with the GP practice by telephone. A composite accessibility score was computed. Predictor variables consisted of patient’s self-reported characteristics, as well as background data about the GP and GP practice from National GP registry. The analysis included multiple linear regression of the composite accessibility score and seven accessibility items. Finally, a qualitative analysis was conducted of free-text survey comments among patients that had a score of 0 (unfavourable) on all the seven accessibility items.

**Results:**

The key factor for patient-experienced accessibility to general practice was seeing their own GP, showing a statistically significant positive correlation (*p*<0.001) across all seven accessibility items and the composite accessibility score in regression analyses. Other associations with positive experience included better self-reported health, and at the GP-level, a specialization in general medicine. Conversely, a negative experience was associated with longer time since the last GP consultation, female patients, and a higher number of GPs at the practice. Qualitative data confirmed accessibility challenges, detailing quantitative scores and highlighted that low accessibility scores were related to difficulties in seeing one’s own GP.

**Conclusions:**

This study highlights the importance of continuity between patient and their GP in improving patients’ experiences of accessibility to general practice. Several GP and GP practice-level factors were related to patient-reported accessibility. These results can be used to inform initiatives aimed at improving accessibility to general practice.

**Supplementary Information:**

The online version contains supplementary material available at 10.1186/s12913-024-11460-8.

## Background

It is a statutory right for all residents in Norway to be registered with a regular General Practitioner (GP), aiming to ensure high-quality, continuous, and coordinated healthcare, at the right time. However, there are challenges related to the GP services with an increasing number of patients on lists without an assigned regular GP. This issue together with an aging and growing older population, means that concerns regarding accessibility are increasing [1, 2]. Similar problems are observed internationally [3], results from the Commonwealth Fund’s international health policy survey indicates that many countries face challenges in providing timely access to appointments [4]. Waller et al. found that patients most often report access to health care as the area that needs improvement in GP practice [5]. The Norwegian government prioritize healthcare access and have introduced regulations to ensure access to GP practice. These regulations mandate that patients receive help within five working days for non-urgent appointments [6].

Access to healthcare is essential for ensuring high quality in health services. However, access is a complex, multidimensional concept that is challenging to measure [7–10]. Levesque et al. proposed a conceptualization of access to healthcare that includes both dimensions of accessibility of systemic characteristics (supply-side) and corresponding individual abilities (demand-side). The supply-side includes: 1) Approachability, referring to how easily healthcare services can be identified and reached; 2) Acceptability, covering cultural and social aspects of healthcare; 3) Availability and accommodation, referring to the physical presence and timely availability of healthcare services and resources; 4) Affordability, addressing the economic capacity; and 5) Appropriateness, ensuring that services meet patients’ needs with the right quality and right time. The demand-side includes: 1) Ability to perceive the need for healthcare; 2) Ability to seek; 3) Ability to reach; 4) Ability to pay; and 5) Ability to engage [7].

In the Norwegian national survey of patients experience with the GP and GP practice from 2021, we found that out of five indicators measuring different aspects of GP practice the lowest indicator score (63 out of 100) was on the patient experience “Accessibility” indicator. The survey included seven items related to accessibility. These items correspond to the supply-side dimensions of accessibility from Levesque et al, specifically “availability and accommodation” and “appropriateness” [7]. Six items address “availability and accommodation” including waiting time for urgent and regular appointments, acceptability of waiting time for urgent and regular appointments, waiting time in the waiting room, and telephone contact, while one question concerns “appropriateness”, whether the GP had enough time with the patient.

Knowledge of the factors associated with patients’ experiences of accessibility can inform improvements for the future organization of GP practices. Previous studies have shown that patient-level predictors such as reason for contact, type of appointment, and frequency of visits are associated with patient-experienced accessibility [11, 12]. Further, patients’ age [11–15], chronic condition [11, 14, 16], income [11, 14], self-reported health [12–14], ethnicity [12–14], and employment status [11, 13] are associated with patient-experienced accessibility. Some of these factors are related to Levesque`s framework of individual capabilities (ability to perceive and to seek) [7]. However, results of previous studies are inconsistent.

Studies on the association between GP and GP practice-level factors and patient-experienced accessibility are relatively scarce. One study indicates that characteristics of GP practices, such as their size, composition, and function, have been identified to represent potential levers for improving patient-experienced accessibility [16]. These GP level variables can be related to the “availability and accommodation” dimension from the Levesque`s framework (related to service capacity, workforce availability and flexibility of service to meet patient’s needs) [7].

In this study, we make an in-depth assessment of factors associated with different aspects of patient-experienced accessibility as measured with the seven accessibility-related items in the Norwegian national survey. Our study supplements previous studies of patient-experienced accessibility, as most studies lack potentially important predictor variables. This includes self-reported health and variables related to continuity like the number of years on the GP’s patient list. Furthermore, we include important predictor variables related to the GP and the GP practice as these might further contribute to understanding patient-experienced accessibility.

The primary aim of this study was to explore the influence of patient-, GP-, and GP practice-level predictor variables on patient-experienced accessibility to GPs and GP practices. Additionally, the study aimed to enhance our understanding of patient-experienced accessibility by analysing free-text comments from patients on a final open-ended questionnaire item. A mixed-methods approach, using both quantitative data and free-text data, enriches the existing literature that predominantly relies on quantitative analysis. The results can enhance our understanding of patient-experienced accessibility and provide valuable insights for developing strategies to improve it.

## Methods

### Setting

The GP scheme was introduced in 2001, designed to ensure that all residents in Norway have access to a regular GP, founded by the National Insurance Scheme. Currently, 98% of Norwegians are registered on a regular GP’s patient list. This GP scheme includes both salaried and self-employed GPs who manage patient lists [17]. The GP practices are in general organized in small units with 2-3 GPs [1]. Normally, there are one or more health secretaries who are usually the first point of contact when booking appointments. Most GPs are self-employed. About 84 percent of GPs operate their practice as a business, while approximately 16 percent are employed on a fixed salary by the municipality as of March 2022 [18].

### Sampling and data collection

This was a secondary analysis of data from a national survey of patient experiences with the GP and GP practice carried out in Norway in 2021-2022. The construction of the patient sample is described elsewhere [19]. Briefly, GP practices were stratified according to the size of the municipality and the number of GPs in the GP practice. Then, 2,000 GPs were randomly selected from within these GP practices. Information about 52 GPs was unavailable. Finally, ten patients were randomly selected from the lists of the 1948 selected GPs, giving a total patient sample of 19,480 [19]. The inclusion criteria were patients aged 16 and older, who had had at least one consultation with their GP during the previous 12 months. Eligible patients registered with an electronic mailbox were sent a digital invitation to respond to the survey through a link. Patients without an electronic mailbox received a letter by post offering the possibility to respond through a link. Non-responders in both groups were sent two reminders by post, including a paper questionnaire.

### The patient experience questionnaire

The questionnaire is a previously validated instrument [20] used in national patient experience surveys in Norway [19, 21]. The questionnaire consists of five subscales measuring different aspects of experiences with the GP and the GP practice, as well as additional items and background questions, resulting in 48 items altogether. The last part of the questionnaire is a free-text field, where the participant could further comment on aspects related to experiences with the GP and the GP’s practice [19].

### Dependent variables

#### Accessibility items

The questionnaire includes seven accessibility related items (Q1-Q7 in Table 1). Five items (Q1-Q5) had a 5-point response format ranging from “not at all”, to “to a very large extent”. The remaining two items (Q6-Q7) on regular waiting time and urgent waiting time for appointments, utilize a different response format based on the number of days waited. See Table 1 for complete questions and original response categories.

**Table 1:** Accessibility items (Q1-Q7), original categories and recoding for composite accessibility score

**Accessibility items**	**Original categories**	**Recoding for composite accessibility score**
Q1. Was the waiting time for an urgent appointment acceptable?	Not at all=1 To a small extent=2 To some extent=3 To a large extent=4 To a very large extent=5	Not at all=0 To a small extent=0 To some extent=0 To a large extent=1 To a very large extent=1
Q2. Was the waiting time for a regular appointment acceptable? (Appointments that are not urgent)		
Q3. Do you feel that the GP has enough time for you?		
Q4. Do you generally have to wait in the waiting room beyond the agreed appointment time?	Not at all=1 To a small extent=2 To some extent=3 To a large extent=4 To a very large extent=5	Not at all=1 To a small extent=1 To some extent=0 To a large extent=0 To a very large extent=0
Q5. Is it difficult to get in touch with your GPs practice by telephone?		
Q6. The last time you needed an appointment with your GP urgent when did you get an appointment?	Same day=1 The next day=2 After 2 days=3 After more than 2 days=4	Same day=1 Next day=1 After 2 days=0 After more than 2 days=0
Q7. How long do you usually have to wait before getting an appointment with your GP? (Regular appointments that are not urgent)	0-1 day=1 2-3 days=2 4-7 days=3 8-14 days=4 More than 14 days=5	0-1 day=1 2-3 days=1 4-7 days=1 8-14 days=0 More than 14 days=0

#### Composite accessibility score

To create a composite accessibility score the responses to Q1-Q7 were coded as “favourable” or “unfavourable” and assigned scores of 1 or 0, respectively. The cut-off in Q1-Q5 was based on the wish to discriminate positive patient experience from neutral and negative experience. The cut-off in Q6-Q7 was based on a requirement for urgent and regular appointments in the Norwegian GP directives [6]. The composite accessibility score was computed based on this coding where all values were summed up, with the final score varying from 0-7, with higher numbers indicating better experience with accessibility. The coding of the items is provided in Table 1.

### Patient-level predictor variables

Background information about the patients was collected through the National GP registry, which included data on patients’ sex, age, and length of time on the GP list. Additionally, self-reported information from the questionnaire included time since last contact with a GP, whether the patients usually see their own GP, education, number of chronic conditions, country of birth, and their physical and mental health. Country of birth was categorized as Norway, Western countries (including Scandinavia, Western Europe, Eastern Europe in EU, North America, and Oceania), and non-Western countries (including Eastern Europe outside the EU, Africa, Asia with Turkey and Central and South America).

### GP and GP practice level predictor variables

Data about the GP and the GP practice were collected through the national GP registry. This included GP’s sex, age, years as a GP, years in the same employment contract, fixed salary, specialization in general practice, list length, available spots on the list, number of GPs at the GP practice, GP group practice, and joint GP list. All patient, GP, and GP practice level predicator variables are provided in Tables 2 and 3. All available variables in the survey at the GP and GP practice-level were included in our analysis. Given the limited evidence on the effect of such variables on patient-experienced accessibility and considering that many of them represent potentially modifiable practice factors, we aimed to test all variables to help contributing to fill this knowledge gap.

**Table 2:** Descriptives of the respondents (*N*=7912)

**Patient level predictor variables**	**Number**	**%**
**Sex**		
Female	4509	57.0
Male	3402	43.0
**Age (years)**	7911	*Mean=58.2 years*
**Education**		
Primary school	1123	14 .5
High school	2744	35.3
University (1-4 years)	2241	28.8
University (more than 4 years)	1662	21.4
**Number of chronic conditions**		
No	2269	29.5
One	2661	34.6
Two	1580	20.5
More than two	1191	15.5
**Birth country**		
Norway	6842	87.3
Western countries	552	7.0
Non-western countries	440	5.6
**Self-reported physical heath**		
Very poor	120	1.5
Rather poor	497	6.3
Both poor and good	2044	26.1
Rather good	3864	49.3
Very good	1305	16.7
**Self-reported mental health**		
Very poor	74	0.9
Rather poor	269	3.4
Both poor and good	1359	17.4
Rather good	3488	44.6
Very good	2630	33.6
**Years the patient has been on GP’s patient list**		*Mean=8.0 years*
Under 1	850	10.7
1-2	1503	19.0
3-4	1097	13.9
5-10	1888	23.9
11 or more	2574	32.5
**Time since last contact with the GP**		
Less than a month	3333	42.3
1-3 months	2426	30.8
4-6 months	1207	15.3
7-12 months	672	8.5
More than 12 months	250	3.2
**Usually meet own GP**		
Yes		88.2
No		11.8

**Table 3:** Descriptives of the GP and GP practice (*N*=1947)

**GP/-GP practice level predictor variables**	**Number**	**% (Mean)**
**GP’s age (years)**	1947	*(Mean=48.8)*
**GP’s sex**		
Female	867	44.5
Male	1080	55.5
**Years in the same employment contract**		*(Mean=10.3)*
**List length**		
0-499	106	5.4
500-999	698	35.9
1000-1499	944	48.5
Over 1500	199	10.2
**Number of available spots on the GP’s list**		
0	511	26.2
1-10	995	51.2
11-99	169	8.7
100 and more	272	14.0
**Number of GPs at the GP practice**		*(Mean=3.9)*
**General medicine specialist**		
Yes	1255	64.5
No	694	35.5
**Fixed salary**		
Yes	281	14.4
No	1666	85.6
**Group practice**		
Yes	1759	90.3
No	188	9.7
**Joint GP list**		
Yes	82	4.2
No	1865	95.8

### Statistical analysis

First, to identify which patient-level predictor variables to include in our models, we conducted a review of the existing literature [11–16, 21, 22]. Second, we conducted descriptive statistical analyses, which included frequencies, percentages, and mean where appropriate on all predictor variables. Third, we conducted bivariate linear regression analysis for all predictor variables both patient-level and GP and GP practice-level with the composite accessibility score as dependent variable. Fourth, because of the sampling method with patients nested within GPs, we evaluated the need for multilevel modelling by estimating the Intraclass Correlation Coefficient (ICC) and the design effect statistics for the seven dependent variables, following the method described by Peugh. We used an estimated design effect above two as an indication of the need for multilevel modelling [23]. We conducted two multiple linear regressions to assess the associations between the composite accessibility score and the two sets of predictor variables: those at a patient-level (Model 1) and those at the GP and GP practice level (Model 2). A full model which included all patient and GP and GP practice variables (Model 3) was also conducted. Last, we conducted 14 multiple linear regressions to assess the associations between each of the seven accessibility items and the same two sets of predictor variables: patient-level and GP and GP practice-level. As the accessibility items are ordinal by nature, we also performed 14 multiple logistic regressions, with a categorization of the accessibility items according to Table 1. A *p*< 0.05 was assigned as the level of statistical significance. Data analyses were performed with IBM SPSS Statistics for Windows, Version 25.0 software (Armonk, NY: IBM Corp.).

### Qualitative analysis

The questionnaire included a free-text field question: “*Feel free to write more about your experiences with your GP and GP practice here*”. The main objective of the qualitative analysis was to get additional information from the patients who had the lowest scores on the accessibility items. We included comments from all patients that scored “0” (unfavourable), with no missing values on all seven accessibility items (*n*=122 comments). Two researchers (EJ & RMN) independently coded these open-ended comments. First, we performed sentiment analysis to address the polarity of the comments (positive, negative, mixed, or neutral), and second, we performed an inductive content analysis to gain insights on the thematic these patients described. Neutral comments (*n*=7) that did not address the GP services or give any specific evaluation of the quality of health care were not included in the content analysis. Hence *n*=115 comments were coded for content. Both researchers performed a meaning condensation of each comment, to capture the essence of what the patient was meant to say in the comment. We included all themes in the analysis, not just those related to accessibility, to get a broad understanding of the patients' experiences. Each open-ended comment was analysed systematically in an iterative manner by creating a preliminary thematic coding structure. When new themes emerged, the coding structure was revised [24]. Throughout the process, the researchers discussed both the themes and the wording that best fitted and described the content. The analysis resulted in five themes.

## Results

The main sample in the study consisted of 18,861 patients after removing people who were unreachable (no digital mailbox, with unknown postal address); who were deceased; and who actively declined to participate. The total number of responses was 7,912, which equated to a response rate of 41.9%.

Of all the respondents, 57% were women, and the average age was 58 years. On average, the respondents had been on the GP's list for eight years and had seven consultations in the previous 24 months. Of all respondents 79% rated their mental health as "rather good" or "very good", and slightly fewer (66%) rated their physical health as "rather good" or "very good". Further, 71% of the respondents had one or more number of chronic conditions and 87% of respondents stated that they were born in Norway. Descriptive statistics for the respondents are presented in Table 2.

The results showed that 65.2% of the patients experienced waiting time for an urgent appointment and 48.3% for regular appointments as acceptable to a large or very large extent. Further, 62.7% of the patients reported getting an urgent appointment the same or the next day. Moreover, 58.2% reported seven days or less regular waiting time for appointments (Table 4).

**Table 4:** Frequencies for accessibility items (Q1-Q7)

**Accessibility items (Q1-Q7)**	**Response category (%)**						
	**Number**	**1**	**2**	**3**	**4**	**5**	**Mean (SD)**
Q1. Was the waiting time for an urgent appointment acceptable? ^a^	6473	7.7	9.3	17.8	29.2	36.0	3.8 (1.2)
Q2. Was the waiting time for a regular appointment acceptable? ^a^	7272	10.0	13.3	28.4	31.3	17.0	3.3 (1.2)
Q3. Do you feel that the GP has enough time for you? ^a^	7778	2.3	6.2	19.9	43.8	27.8	3.9 (1.0)
Q4. Do you generally have to wait in the waiting room beyond the agreed appointment time? ^a^	7734	5.5	33.3	36.1	15.5	9.8	2.9 (1.0)
Q5. Is it difficult to get in touch with your GPs practice by telephone? ^a^	7586	35.8	31.4	22.8	6.5	3.5	2.1 (1.1)
Q6. The last time you needed an appointment with your GP urgent, when did you get an appointment? ^b^	6486	38.7	24.0	12.9	24.4	-	2.2 (1.2)
Q7. How long do you usually have to wait before getting an appointment with your GP? ^c^	7304	8.6	20.0	29.6	25.6	16.2	(1.2)

^a^1=Not at all, 2= To a small extent, 3= To some extent, 4=To a large extent, 5=To a very large

^b^1=same day; 2=next day; 3=after 2 days; 4=after more than 2 days

^c^1=0-1 day; 2= 2-3 days;3=4-7 days; 4= 8-14 days; 5= more than 14 days

The ICC ranged from 0.08 (Q1 and Q6) to 0.26 (Q7). None of the seven accessibility items had a design effect above two, which supported the decision of conducting linear regressions without accounting for nested (multilevel) design.

### Multiple linear regression with the composite accessibility score

#### Patient-level predictor variables – Model 1

A statistically significant positive association was observed between the composite accessibility score and patients who usually meet their own GP (B=1.165, *p*<0.001), had an increased number of years on GPs list (B=0.023, *p*<0.001), better self-reported mental (B=0.193, *p*<0.001) and physical health (B=0.133, *p*<0.001), and were born in a Western country other than Norway (B=0.262, *p*=0.003). Conversely, a negative association with the composite accessibility score was found for female patients (B=-0.237, *p*<0.001), and those with longer time since last contact with the GP (7-12 months B=-0.481, *p*<0.001) (Table 5).

**Table 5:** Results of linear regression analysis with the composite accessibility score as dependent variable

**Predictor variables**	**Bivariate regression**		**Multiple regression, model 1 – Patient level**		**Multiple regression, model 2 – GP level**		**Multiple regression, model 3 – Full model**	
	**B (95% CI)**	**Beta**	**B (95% CI)**	**Beta**	**B (95% CI)**	**Beta**	**B (95% CI)**	**Beta**
***Patient level***								
**Sex**								
(Male. RG)	-0.282*** (-0.369, -0.194)	**-0.071**	-0.237*** (-0.325, -0.149)	**-0.059**			-0,227*** (-0.316, -0.137)	**-0.057**
Female								
**Age**	0.007*** (0.005, 0.010)	**0.064**	0.002 (-0.001, 0.004)	0.013			0,002 (-0.001, 0.004)	0.013
**Education**								
Primary school (RG)								
University (more than 4 years)	-0.047 (-0.197, 0.102)	-0.010	-0.148 (-0.302, 0.006)	-0.031			-0,133 (-0.287, 0.021)	-0.028
**Number of chronic conditions**								
No (RG)								
One	-0.021 (-0.132, 0.090)	-0.005	-0.008 (-0.122, 0.106)	-0.002			-0,013 (-0.127, 0.100)	-0.003
Two	-0.092 (-0.219, 0.035)	-0.019	-0.012 (-0.147, 0.124)	-0.002			-0,015 (-0.150, 0.120)	-0.003
More than two	-0.131 (-0.270, 0.008)	-0.024	0.000 (-0.158, 0.159)	0.000			-0,003 (-0.161, 0.154)	-0.001
**Country of Birth**								
Norway (RG)								
Western countries	0.131 (-0.040, 0.302)	0.017	0.262** (0.091, 0.432)	**0.034**			0,227** (0.057, 0.396)	**0.029**
Non- Western countries	-0.182 (-0.372, 0.008)	-0.021	0.039 (-0.154, 0.233)	0.005			0,027 (-0.166, 0.220)	0.003
**Self-reported physical heath**	0.173*** (0.122, 0.223)	**0.076**	0.133*** (0.071, 0.196)	**0.059**			0,133*** (0.071, 0.195)	**0.058**
**Self-reported mental health**	0.263*** (0.213, 0.314)	**0.114**	0.193*** (0.136, 0.251)	**0.084**			0,198*** (0.141, 0.255)	**0.086**
**Years on GP list**	0.035*** (0.029, 0.041)	**0.124**	0.023*** (0.017, 0.030)	**0.083**			0,016** (0.006, 0.027)	**0.058**
**Time since last contact with the GP**								
Less than a month (RG)								
1-3 months	-0.072 (-0.175, 0.031)	-0.017	-0.123** (-0.226, -0.020)	**-0.029**			-0,127** (-0.229, -0.025)	**-0.030**
4-6 months	-0.102 (-0.231, 0.028)	-0.019	-0.187** (-0.318, -0.056)	**-0.034**			-0,186** (-0.316, -0.056)	**-0.034**
7-12 months	-0.375*** (-0.538, -0.212)	**-0.053**	-0.481*** (-0.646, -0.316)	**-0.068**			-0,489*** (-0.653, -0.325)	**-0.069**
More than 12 months	-0.772*** (-1.027, -0.517)	**-0.068**	**-0.550 (-0.817, -0.284)**	**-0.046**			-0,532*** (-0.797, -0.267)	**-0.045**
**Usually meet own GP**	1.284*** (1.152, 1.417)	**0.210**	1.165*** (1.300, 1.029)	**0.191**			1,111*** (0.973, 1.249)	**0.182**
***GP/GP practice level***								
**GPs age**	0.016*** (0.013, 0.020)	**0.095**			-0.003 (-0.010, 0.003)	-0.020	-0.006 (-0.012, 0.001)	-0.033
**GPs sex**								
Male (RG)								
Female	-0.266*** (-0.354, -0.179)	**-0.067**			-0.188*** (-0.278, -0.097)	**-0.047**	-0.100*(-0.192, -0.008)	**-0.025**
**Years in the same employment contract**	0.029*** (0.023, 0.034)	**0.108**			0.015** (0.005, 0.025)	**0.057**	0.002 (-0.010, 0.015)	0.009
**List length**								
0-499 (RG)								
500-999	0.107 (-0.102, 0.316)	0.026			-0.095 (-0.315, 0.125)	-0.023	-0.154 (-0.378, 0.071)	-0.037
1000-1499	0.219* (0.014, 0.425)	**0.056**			-0.226 (-0.461, 0.008)	-0.057	-0.270* (-0.509, -0.031)	**-0.068**
Over 1500	0.314** (0.073, 0.556)	**0.047**			-0.294** (-0.563, -0.025)	**-0.044**	-0.309*(-0.582, -0.035)	**-0.046**
**Number of available spots on the GPs list**								
0 (RG)								
1-10	-0.032 (-0.135, 0.071)	-0.008			-0.017 (-0.120, 0.087)	-0.004	0-039 (-0.065, 0.142)	0.010
11-99	-0.195* (-0.372, -0.018)	**-0.026**			-0.235** (-0.414, -0.056)	**-0.032**	-0.099 (-0.279, 0.080)	-0.013
100 and more	0.010 (-0.134, 0.154)	0.002			-0.088 (-0.246, 0.069)	-0.015	-0.035 (-0.193, 0.122)	-0.006
**Number of GPs at the GP practice**	-0.092*** (-0.114, -0.070)	**-0.092**			-0.089*** (-0.113, -0.065)	**-0.089**	-0.085***(-0.109, -0.061)	**-0.084**
**General medicine specialist**								
No (RG)								
Yes	0.484*** (0.392, 0.576)	**0.116**			0.380*** (0.274, 0.487)	**0.091**	0.316***(0.209, 0.424)	**0.076**
**Fixed salary**								
No (RG)								
Yes	-0.412*** (-0.541, -0.284)	**-0.071**			-0.348*** (-0.499, -0.196)	**-0.060**	-0.176*(-0.329, -0.023)	**-0.030**
**GP group practice**								
No (RG)								
Yes	-0.421*** (-0.575, -0.268)	**-0.060**			-0.284*** (-0.443, -0.125)	**-0.041**	-0.247*(-0.407, -0.088)	**-0.035**
**Joint GP list**								
No (RG)								
Yes	0.030 (-0.191, 0.250)	0.003			0.209 (-0.021, 0.440)	0.021	0.248*(0.017, 0.479)	**0.025**
***Model Statistics***								
* R* ^2^	-		0.075		0.032		0.090	
Adjusted R^2^	-		0.073		0.030		0.086	

*CI *Confidence interval, *= *p*<0.05*,  **= *p*<0.01, ***=*p* <0.001 (significant estimates in bold). The composite score scale is from 0-7, with higher numbers indicating better patient-experience accessibility

#### GP and GP practice level predictor variables – Model 2

A statistically significant positive association was observed between the composite accessibility score and GPs with increased number of years in the same employment contract (B=0.015, *p*=0.004), and those specialized in general medicine (B=0.380, *p*<0.001). A negative association with the composite accessibility score was found for GPs in group practices (B=-0.284, *p*<0.001), fixed salaries (B=-0.348, *p*<0.001), female sex (B=-0.188, *p*<0.001), lists length over 1500 patients (B=-0.294, *p*=0.032), 11-99 available spots on the list (B=-0.235, *p*=0.010), and increased number of GPs at the GP practice (B=-0.089, *p*<0.001) (Table 5).

#### Full model including both patient and GP and GP practice variables – Model 3

In the full model (Model 3), when controlled for patient-level variables, the GP-level variables “years in the same employment contract” and “number of available spots” are no longer statistically significant. The GP practice level variable “joint GP list” is statistically significant when controlled for patient-level variables (Table 5).

Model 1 explains a significant portion of the variance in the composite accessibility score (R^2^ = 0.075). Model 2 contributes less (R^2^ = 0.032). The full model (Model 3), combining all variables, provides the best model fit (R^2^ = 0.090) 

### Multiple linear regression between patient- level predictor variables and single items of accessibility

*Usually meeting own GP and time on GP list:* “Usually meeting own GP” had an association with positive patient experience of all seven accessibility items. Further, being on a GP's list for more years was associated with the experience of reduced waiting times both for regular and urgent appointments, increased acceptance of urgent waiting times, less waiting time in the waiting room, less difficulty in getting in touch with the GPs practice as well as having enough time with the GP. *Self-reported mental and physical health:* Better self-reported mental health was associated with better experience of waiting time for urgent appointments. Better self-reported mental and physical health was associated with acceptance of urgent and regular waiting time. In addition, having better self-reported mental and physical health had an association with a patient experience of less waiting time in the waiting room, enough time with the GP, and less difficulty getting in touch with the GP practice via telephone (Table 6). *Patient sex:* Female patients, despite reporting shorter waiting time for non-urgent appointments, showed a lower acceptance of this waiting time. However, there was an association between female sex and better experience with urgent waiting time and acceptance of this waiting time. Female patients experienced longer waiting time in the waiting room and getting in touch with the GP practice via telephone was more difficult compared to men. *Intervals on the last time since contact with GP:* Patients who have not seen their GP in the last 7-12 months or more experienced longer waiting time for urgent appointments, as well as experiencing waiting times for urgent and regular appointments as less acceptable. Longer time since last contact with the GP was associated with negative experience of having enough time with GP as well as more difficulty of getting in touch with GP practice via telephone. *Birth country:* There were no statistically significant associations between country of birth and waiting time for urgent appointments. However, patients who were born in other countries than Norway had lower acceptance of this waiting time. Turning to waiting time for regular appointments, patients born in other country than Norway reported longer waiting times, compared to patients born in Norway. However, only patients born in non-Western countries found this waiting time less acceptable. Patients from other countries than Norway had more positive experiences with the waiting time in the waiting room and getting in touch with the GP practice via telephone. Being born in other Western countries, compared to Norway, was also positively associated with the experience of having enough time with the GP. *Self-reported chronic condition:* One or more chronic conditions were associated with a longer waiting time for appointments, with no associations related to the acceptance of the waiting time compared to those with no chronic condition. There was no difference between chronic conditions and waiting time for urgent appointments, and patients with chronic conditions tend to find the waiting time more acceptable compared to patients with no chronic condition. Patients with chronic conditions reported more negative experiences with waiting time in the waiting room as well as more difficulty in getting in touch with the GP practice via telephone. *Patient age:* older patients experienced longer waiting time for urgent appointments and lower acceptance of this waiting time. However, higher age had a positive association with regular waiting time, and acceptance of this waiting time. Higher age had also positive association with less waiting time in the waiting room and less difficulty in getting in touch with the GP practice via telephone (see Table 6 for details on effect sizes).

**Table 6:** Multiple linear regression with the seven accessibility items as the dependent variable and patient-level variables as predictors

**Dependent variables**	**Waiting time for an urgent appointment (*n*= 5728)**		**Waiting time for an urgent appointment acceptable (*n*= 5726)**		**Regular waiting time for an appointment (not urgent) (*n*= 6469)**		**Waiting time for a regular appointment acceptable (not urgent) (*n*= 6443)**		**Enough time with the GP (*n*= 6864)**		**Waiting time in the waiting room (*n*= 6842)**		**Difficulty in getting in touch with your GPs practice by telephone** **(*n*= 6706)**	
**Predictor variables**	**B (95% CI)**	**Beta**	**B (95% CI)**	**Beta**	**B (95% CI)**	**Beta**	**B (95% CI)**	**Beta**	**B (95% CI)**	**Beta**	**B (95% CI)**	**Beta**	**B (95% CI)**	**Beta**
**Sex**														
Male (RG)														
Female	-0.130*** (-0.190, -0.070)	**-0.054**	0.101*** (0.039, 0.162)	**0.040**	0.334*** (-0.004, -0.001)	**0.140**	-0.068** (-0.124, -0.012)	**-0.028**	-0.041 (0.083, 0.002)	-0.021	0.167*** (0.120, 0.214)	**0.079**	0.097*** (0.047, 0.146)	**0.045**
**Age**	0.004*** (0.002, 0.006)	**0.054**	-0.007*** (-0.009, -0.005)	**-0.093**	-0.002** (-0.083, 0.090)	**-0.033**	0.002** (0.000, 0.004)	**0.026**	0.000 (-0.001, 0.002)	0.005	-0.013*** (-0.014, -0.011)	**-0.210**	-0.006*** (-0.008, -0.005)	**-0.098**
**Education**														
Primary school (RG)														
High school	-0.073 (0.165, 0.020)	-0.029	0.101** (0.006, 0.195)	**0.039**	0.004 (0.039, 0.218)	0.001	0.024 (-0.063, 0.112)	0.010	-0.029 (-0.096, 0.037)	-0.014	-0.037 (-0.110, 0.037)	-0.017	0.047 (-0.030, 0.124)	0.021
University (1-4 years)	-0.068 (-0.165, 0.029)	-0.025	0.221*** (0.122, 0.319)	**0.081**	0.128** (0.139, 0.332)	**0.049**	0.131** (0.040, 0.221)	**0.050**	-0.011 (-0.080, 0.058)	-0.005	0.020 (-0.056, 0.096)	0.009	0.094** (0.014, 0.174)	**0.040**
University (more than 4 years)	-0.052 (-0.156, 0.053)	-0.018	0.254*** (0.148, 0.360)	**0.084**	0.235*** (0.063, 0.206)	**0.082**	0.139** (0.041, 0.236)	**0.048**	-0.040 (-0.115, 0.034)	-0.017	0.068 (-0.013, 0.150)	0.027	0.113** (0.027, 0.200)	**0.043**
**Number of chronic conditions**														
No (RG)														
One	0.022 (-0.056, 0.100)	0.009	0.086** (0.007, 0.165)	**0.033**	0.135*** (0.112, 0.282)	**0.054**	-0.016 (-0.088, 0.056)	-0.006	-0.032 (-0.087, 0.023)	-0.016	0.051 (-0.009, 0.112)	0.023	0.093** (0.029, 0.157)	**0.041**
Two	0.024 (-0.069, 0.117)	0.008	0.131** (0.036, 0.225)	**0.042**	0.197*** (0.120, 0.319)	**0.068**	0.012 (-0.074, 0.097)	0.004	0.023 (-0.043, 0.088)	0.010	0.090** (0.018, 0.162)	**0.035**	0.038 (-0.039, 0.114)	0.014
More than two	-0.027 (-0.134, 0.081)	-0.008	0.135** (0.027, 0.244)	**0.040**	0.220*** (-0.115, 0.014)	**0.067**	0.053 (-0.047, 0.154)	0.016	0.011 (-0.066, 0.087)	0.004	0.084** (0.000, 0.168)	**0.029**	0.077 (-0.012, 0l166)	0.026
**Country of Birth**														
Norway (RG)														
Western countries	0.064 (-0.050, 0.179)	0.014	-0.199*** (-0.316, -0.082)	**-0.042**	-0.373*** (-0.688, -0.448)	**-0.081**	-0.030 (-0.137, 0.077)	-0.006	0.111** (0.029, 0.193)	**0.030**	-0.150*** (-0.240, -0.060)	**-0.037**	-0.193*** (-290, -0.096)	**-0.046**
Non- Western countries	0.102 (-0.024, 0.227)	0.020	-0.531*** (-0.658, -0.404)	**-0.102**	-0.568*** (-0.054, 0.025)	**-0.110**	-0.270***(-0.391, -0.150)	**-0.052**	-0.051 (-0.144, 0.042)	-0.012	-0.171*** (-0.275, -0.068)	**-0.037**	-0.140** (-0.249, -0.032)	**-0.030**
**Self-reported physical heath**	-0.018 (-0.060, 0.025)	-0.013	0.092*** (0.049, 0.135)	**0.064**	-0.015 (-0.057, 0.014)	-0.011	0.106*** (0.066, 0.145)	**0.076**	0.115*** (0.084, 0145)	**0.103**	-0.044** (-0.078, -0.011)	**-0.037**	-0.047** (-0.082, -0.012)	**-0.038**
**Self-reported mental health**	-0.060** (-0.099, -0.021)	**-0.043**	0.097*** (0.058, 0.136)	**0.067**	-0.021 (-0.006, 0.002)	-0.015	0.089*** (0.053, 0.125)	**0.064**	0.138*** (0.110, 0.165)	**0.123**	-0.085*** (-0.116, -0.055)	**-0.070**	-0.072*** (-0.104, -0.040)	**-0.057**
**Years on GP list**	-0.016*** (-0.020, -0.011)	**-0.091**	0.017*** (0.012, 0.021)	**0.095**	-0.002 (0.268, 0.437)	-0.010	0.015*** (0.011, 0.019)	**0.086**	0.011*** (0.008, 0.014)	**0.081**	0.004*** (0.000, 0.007)	**0.024**	-0.008*** (-0.011, -0.004)	**-0.051**
**Time since last contact with the GP**														
Less than a month (RG)														
1-3 months	0.048 (-0.022, 0.117)	0.018	-0.041 (-0.112, 0.030)	-0.015	-0.050 (-0.066, 0.098)	-0.020	-0.026 (-0.091, 0.038)	-0.010	-0.054** (-0.103, -0.004)	**-0.026**	0.016 (-0.039, 0.070)	0.007	0.048 (-0.010, 0.105)	0.020
4-6 months	0.092** (0.003, 0.181)	**0.028**	-0.024 (-0.114, 0.067)	-0.007	0.016 (-0.018, 0.190)	0.005	-0.027 (-0.110, 0.055)	-0.008	-0.147*** (-0.210, -0.083)	**-0.055**	0.057 (-0.013, 0.127)	0.020	0.123** (0.050, 0.197)	**0.041**
7-12 months	0.324*** (0.205, 0.442)	**0.071**	-0.199*** (-0.318, -0.080)	**-0.043**	0.086 (-0.134, 0.214)	0.020	-0.118** (-0.223, -0.014)	**-0.027**	-0.170*** (-0.249, -0.090)	**-0.049**	0.008 (-0.079, 0.096)	0.002	0.132** (0.039, 0.225)	**0.034**
More than 12 months	0.219** (0.039, 0.400)	**0.031**	-0.169 (-0.352, 0.014)	-0.023	0.040 (-0.479, -0.267)	0.005	-0.186** (-0.361, -0.010)	**-0.025**	-0.273*** (-0.401, -0.145)	**-0.048**	0.066 (-0.076, 0.207)	0.010	0.194** (0.042, 0.346)	**0.030**
**Usually meet own GP**	-0.446*** (-0.356, -0.537)	**-0.123**	0.622*** (0.713, 0.530)	**0.166**	-0.352*** (-0.004, -0.001)	**-0.097**	0.655*** (0.740, 0.570)	**0.178**	0.642*** (0.707-0.576)	**0.216**	-0.429*** (-0.357, -0.501)	**0.132**	-0.439*** (-0.363, -0.515)	**-0.132**

*CI* Confidence interval, *= *p*<0.05*; **= *p*<0.01; ***= *p*<0.001 (statistically significant estimates in bold)

### Multiple linear regression between GP and GP practice-level predictor variables and single items of accessibility

*General medicine specialist and years under the same contract:* Having a GP specialized in general medicine had a statistically significant positive association with patient experience on all seven accessibility items. Similar results are shown with GPs who have more years under the same contract. *Fixed salary:* Having a GP on a fixed salary was associated with negative patient experience with all accessibility items except for waiting time in the waiting room and difficulty of getting in touch with the GP practice via telephone. *Available spots on the GP list, number of GPs at the GP practice, and list length:* A higher number of available spots on the GP's list and a higher number of GPs at the GP practice were associated with patient`s experience of -longer waiting times for an urgent appointment, -longer waiting time in the waiting room, and -more difficulty of getting in touch with the GPs practice via telephone. In addition, a higher number of available spots on the GP's list as well as GPs having a longer list length was associated with negative patient experience of having enough time with the GP. A higher number of GPs at the GP practice was associated with a longer waiting time for regular appointment and negative experience related to acceptance of this waiting time (see Table 7 for details on effect sizes).

**Table 7:** Multiple linear regression with the seven accessibility items as the dependent variable and GP and GP practice level variables as predictors

**Dependent variables**	**Waiting time for an urgent appointment (*n*= 5728)**		**Waiting time for an urgent appointment acceptable (*n*= 5726)**		**Regular waiting time for an appointment (not urgent) (*n*= 6469)**		**Waiting time for a regular appointment acceptable (not urgent) (*n*= 6443)**		**Enough time with the GP (*n*= 6864)**		**Waiting time in the waiting room (*n*= 6842)**		**Difficulty in getting in touch with your GPs practice by telephone (*n*= 6706)**	
**Predictor variables**	**B (95% CI)**	**Beta**	**B (95% CI)**	**Beta**	**B (95% CI)**	**Beta**	**B (95% CI)**	**Beta**	**B (95% CI)**	**Beta**	**B (95% CI)**	**Beta**	**B (95% CI)**	**Beta**
**GPs age**	0.003 (-0.002, 0.007)	0.025	-0.001 (0.006, 0.004)	-0.009	0.001 (-0.003, 0.005)	0.007	-0.001 (-0.005, 0.003)	-0.009	-0.002 (-0.005, 0.001)	-0.025	0.003 (-0.001, 0.006)	0.027	0.004** (0.045, 0.001)	**0.008**
**GPs sex**														
Male (RG)														
Female	-0.010 (-0.071, 0.051)	-0.004	0.006 (-0.058, 0.069)	0.002	0.198*** (0.142, 0.254)	**0.083**	-0.121*** (-0.178, -0.064)	**-0.050**	-0.004 (-0.049, 0.041)	-0.002	0.085*** (0.134, 0.040)	**0.040**	0.056** (0.026, 0.005)	**0.106**
**Years in the same employment contract**	-0.010** (-0.017, -0.003)	**-0.062**	0.011** (0.004, 0.018)	**0.066**	0.001 (-0.005, 0.008)	0.009	0.009** (0.002, 0.015)	**0.054**	0.012***(0.007, 0.017)	**0.095**	0.000 (-0.005, 0.006)	0.003	-0.010*** (-0.069, -0.016)	**-0.004**
**List length**														
0-499 (RG)														
500-999	-0.017 (-0.166, 0.132)	-0.007	0.048 (-0.106, 0.203)	0.019	0.150** (0.013, 0.286)	**0.061**	-0.033 (-0.172, 0.106)	-0.013	0.004 (-0.105, 0.114)	0.002	0.097 (-0.023, 0.217)	0.044	0.139** (0.062, 0.016)	**0.261**
1000-1499	0.002 (-0.157, 0.160)	0.001	-0.011 (-0.175, 0.153)	-0.005	0.112 (-0.034, 0.257)	0.047	-0.085 (-0.233, 0.063)	-0.036	-0.071 (-0.188, 0.045)	-0.037	0.148** (0.021, 0.276)	**0.071**	0.254*** (0.118, 0.124)	**0.385**
Over 1500	0.035 (-0147, 0.217)	0.009	-0.016 (-0.205, 0.173)	-0.004	-0.014 (-0.182, 0.153)	-0.004	-0.098 (-0.268, 0.072)	-0.024	-0.194** (-0.328, -0.061)	**-0.060**	0.154** (0.007, 0.300)	**0.043**	0.273*** (0.074, 0.123)	**0.424**
**Number of available spots on the GPs list**														
0 (RG)														
1-10	0.047 (-0.022, 0.117)	0.020	-0.012 (-0.085, 0.060)	-0.005	-0.055 (-0.119, 0.009)	-0.023	0.040 (-0.025, 0.106)	0.017	-0.027 (-0.078, 0.024)	-0.014	-0.012 (-0.067, 0.044)	-0.006	0.020 (0.009, -0.037)	0.078
11-99	0.247*** (0.125, 0.368)	**0.055**	-0.178** (-0.303, -0.052)	**-0.038**	-0.161** (-0.272, -0.051)	**-0.037**	-0.009 (-0.121, 0.0104)	-0.002	-0.218*** (-0.306, -0.130)	**-0.061**	0.127** (0.030, 0.223)	**0.033**	0.151** (0.038, 0.051)	**0.250**
100 and more	0.123** (0.016, 0.229)	**0.035**	-0.047 (-0.158, 0.063)	-0.013	-0.302*** (-0.400, -0.205)	**-0.088**	0.048 (-0.052, 0.147)	0.014	-0.179*** (-0.257, -0.101)	**-0.064**	0.041 (-0.045, 0.126)	0.013	0.070 (0.023, -0.018)	0.158
**Number of GPs at the GP practice**	0.018** (0.001, 0.034)	**0.029**	-0.009 (-0.026, 0.008)	-0.014	0.058*** (0.043, 0.072)	**0.096**	-0.049*** (-0.064, -0.034)	**-0.081**	-0.010 (-0.022, 0.002)	-0.021	0.025*** (0.012, 0.038)	**0.047**	0.051*** (0.093, 0.038)	**0.064**
**General medicine specialist**														
No (RG)														
Yes	-0.185*** (-0.257, -0.112)	**-0.072**	0.223*** (0.148, 0.298)	**0.084**	-0.104** (-0.170, -0.037)	**-0.041**	0.195*** (0.128, 0.263)	**0.077**	0.098*** (0.045, 0.150)	**0.048**	-0.130*** (-0.187, -0.072)	**-0.059**	-0.202*** (-0.089, -0.261)	**-0.142**
**Fixed salary**														
No (RG)														
Yes	0.189*** (0.086, 0.292)	**0.053**	-0.210*** (-0.316, -0.104)	**-0.057**	0.254*** (0.160, 0.347)	**0.072**	-0.253*** (-0.349, -0.158)	**-0.072**	-0.089** (-0.164, -0.014)	**-0.031**	-0.069 (-0.151, 0.013)	-0.022	0.066 (0.021, -0.018)	0.150
**Group GP practice**														
No (RG)														
Yes	-0.012 (-0.118, 0.094)	-0.003	0.011 (-0.100, 0.121)	0.002	0.207*** (0.109, 0.305)	**0.050**	-0.083 (-0.184, 0.017)	-0.020	-0.057 (-0.136, 0.021)	-0.017	0.148*** (0.062, 0.234)	**0.040**	0.074 (0.020, -0.014)	0.162
**Joint GP list**														
No (RG)														
Yes	-0.011 (-0.168, 0.146)	-0.002	0.050 (-0.112, 0.212)	0.008	0.003 (-0.140, 0.145)	0.000	0.016 (-0.129, 0.162)	0.003	-0.006 (-0.120, 0.107)	-0.001	-0.128** (-0.253, -0.004)	-0.024	-0.212*** (-0.039, -0.339)	**-0.084**

*CI *Confidence interval; *= *p*<0.05*; **= *p*<0.01; ***=*p*<0.001 (statistically significant estimates in bold).

Longer list length was also associated with negative patient experience for the waiting time in the waiting room and getting in touch with the GP`s practice via telephone. *GPs sex:* female GPs were associated with negative patient experiences in four of the seven accessibility items: longer waiting time for regular appointments, acceptance of this waiting time, waiting time in the waiting room, and difficulty of getting in touch with the GPs practice via telephone (Table 7). The results from the logistic regressions are provided in appendix 1 (Supplemental Table 1 and 2).

### Qualitative analysis

Most of the comments (87.8%) were negative, some comments were mixed containing both positive and negative content (11.3%), and only a small proportion (0.9%) of the comments were positive. 61% of comments were directly connected to the accessibility. We divided the comments into five themes:

#### Waiting time

In the theme related to waiting times patients mostly focused on long or inflexible waiting times, both for urgent appointments and regular appointments. Another common topic was time spent in waiting rooms. Some patients also commented that they used private health services due to the difficulty of accessing a GP and long waiting times.

#### Referral

Referral was mostly about the patients not experiencing being referred further and taken seriously concerning their health problems they presented to the GP. This created insecurity. Some patients also mentioned that there could be a long waiting time to be referred and that this delayed the relevant diagnostics and treatment. For example, one participant wrote: *“I`m not taken seriously. Not treated with respect. The GP refused to refer me to an orthopaedist. I got a referral from a substitute, which resulted in a knee prosthesis. The GP refused to give me pain medication after the knee surgery. I had to “get myself together” he said, because he didn’t think that I had any pain”.*

#### Consultation time

Time used in consultation and the experience of a busy and stressed GP were common topics for the patients. Some patients commented that they could not talk about more than one health problem with the GP and were told to book another appointment for other additional problems.

#### Getting through by telephone

This was a common topic. Many patients found it very difficult, or sometimes impossible, to get in touch with the GP’s practice by telephone.

#### Digital communication

Some patients made comments about digital communication and their difficulties in booking an appointment online.

An example of comments about consultation time and referral is: *“I feel my GP try to end the consultation as soon as possible. When I have a health problem, I either get medication or I am told it will pass by itself. I never get a referral for a medical examination for my health problems. I am therefore considering changing the GP when the waiting lists are a bit less for other GPs”.*

Another example of comments related to accessibility via telephone, communication, consultation time as well as waiting time for appointments, and digital communication is: *“It is almost impossible to get through the telephone! The GP has a great workload and calls me after 6.30 p.m. The communication between me and the GP has been ongoing via Helsenorge.no* [Norwegian digital health services] *to 11 p.m. There is a long waiting time for a regular appointment – up to 14 days. There are also delays on urgent appointments, where I must wait up to 2 hours. He is busy and there is little time in the consultation, and sometimes I must book a new consultation to get answers for other additional problems. I experience that it can be difficult to get a referral. I miss communication via an app on the telephone... The GP practice is only using Helsenorge.no, which is cumbersome, they have stopped using SMS”.*

A few commented on the long travel routes to the GP practice, making accessibility to care more challenging, especially due to the waiting times at the practice.

Other important themes among patients who reported unfavourable scores of accessibility included continuity of care, communication with the GP, and professionalism. Patients commented on not being able to see their own GP, or not having a GP, and hence often seeing substitute doctors. The absence of their own GP created a lack of continuity, which could also make the patients feel unsafe*.* As a further consequence, many patients experienced a lack of comprehensive follow-up.

## Discussion

Several patient, GP, and GP practice-level predictor variables were associated with patient-experienced accessibility. The patient-level predictor variable with the strongest association with the accessibility composite score was seeing own GP. Other patient-level variables that had a strong association with the composite score were years on GPs list and self-reported mental health. In addition, the GP- and GP practice-level predictors that had a strong association with the composite score were specialization in general medicine, and number of GPs at the GP practice.

Analysis of free-text comments from patients with poor accessibility scores confirmed accessibility challenges for this group, detailing and contextualizing quantitative scores concerning waiting time for both urgent and regular appointments, time with the GP, and getting through the telephone. These qualitative results provided additional accessibility topics such as digital communication and geographical distance, making accessibility to care more challenging. These qualitative results touch upon the framework of Levesque, not only concerning the demand-side factors such as “ability to reach”, but also “ability to perceive” and “to engage”. Qualitative results are also related to the supply-side dimensions such as “availability and accommodation”, “appropriateness”, and “acceptability” [7]. Additionally, an important theme was connected to continuity of care, more specifically not being able to see their own GP, or not having a GP, and hence often seeing substitute doctors. These qualitative results are also coherent with the main results from regression analysis, showing that low accessibility scores were associated to difficulties in seeing one’s own GP.

Similar results were found in previous research; self-reported continuity of care is strongly associated with higher patient satisfaction and studies suggests that improving continuity of care may improve patient satisfaction with the GP as well as with their GP practice [25, 26]. Another recent study by Cook et al. showed that when GPs improved their appointment delay, continuity increased, patients’ use of the emergency department decreased [27]. In addition, Stephen et al. found that less satisfied patients had longer waiting times for appointments and reduced continuity with a specific GP [28]. Furthermore, according to patients’ perspective in the UK, satisfactory standards for accessibility and continuity in GP practice include being able to get an appointment the next day, no longer than a 6–10-minute wait for consultations to begin and seeing the same GP “a lot of the time”. The results from the study show that continuity is associated with accessibility [29]. Results from the international QUALICOPC study, which includes Norway, show that accessibility in primary health care was secondary to good communication. However, in the same study the accessibility item “‘I can get an appointment easily’’ was regarded as important or very important by 99% of Norwegian patients. Similarly, more than 90% of patients from all the other Nordic countries included in the study except Denmark also regarded this item as either important or very important [30].

In our study, we also found that the more years the patient had been on their GP’s list, the more positive were the patient’s experiences with accessibility. This may suggest that continuity is not only important but also connected to the experience of good accessibility. In a priority setting, there is an ongoing discussion about what is most important, access to care or continuity of care [31, 32]. Consequently, there are an increasing number of patient trade-off studies exploring continuity and access in primary care, using methods such as discrete choice experiments. Oliver et al. found that even though timely access to the GP seemed to be the leading priority for patients, the relative importance of continuity was great – especially in the context of a routine check-up, compared to a cold or sudden pain [31]. Another trade-off study shows that the speed of accessibility is of limited importance to patients and, for many, outweighed by choice of GP or convenience of appointment [32]. Studies show that continuity is a valued element in primary care – by both patients and GPs. It is associated with quality of care and seems to especially benefit older patients with complex conditions, in addition, it may reduce hospital admission rates [33–37]. Based on previous studies and this study, both accessibility and continuity of care should be prioritized and included in future improvements of GP practice.

Norway’s regular GP scheme has been under pressure for a long time and is in a serious situation due to the lack of GPs [2]. Similar challenges have been described in other countries like the UK [3]. There is particular concern for patients with complex health problems, including those with chronic conditions and multimorbidity, who are major users of the GP and the GP practice [2, 3, 38]. These patients seem to be particularly vulnerable if they are on a list without a permanent GP, experience poorer availability of a GP, or are in a situation where their GP is constantly changing [2]. Studies have suggested that enhanced accessibility and continuity are associated with better self-reported physical and mental health [39, 40]. Our study showed that better self-reported physical and mental health are associated with better experience of accessibility. This is similar to the results of a recent study into British GP practice, exploring the association between patients’ self-reported health, clinical quality, and patient-reported satisfaction with accessibility and GP consultations. The results showed that better self-reported health was positively associated with the patient satisfaction of accessibility [41]. Previous research also shows that patients with worse mental health are more likely to experience multiple barriers to accessibility both before, and especially after, they reach primary health care [14].

Rapid access is often balanced against a greater involvement in the consultation when seeing specific GPs. This is particularly valued by the patients who have multimorbidity, including psychological problems [42]. Our results show that patients with chronic conditions experience longer waiting time to get a regular appointment, which is also reflected in another similar study [11]. In addition to this, our results from analysis of the free-text comments show that patients who experience poor accessibility comment on having a poor follow-up –as well as having a lack of continuity. This may also make the patients feel “unsafe”.

Other findings in this study show that women had significantly more negative experiences with accessibility than men. This is reflected in another international study exploring barriers to accessibility to GP practice [14]. Other studies have found that women seek physical and mental health care more often than men, and that women report having had longer consultation times [43–45]. In the same way, women's overall satisfaction with visits to the GP is more dependent than men's on informational content, continuity of care, and multidisciplinarity [46]. Some of the results related to high expecations among women may explain the results of our study.

Results from our study related to the composite accessibility score show positive experience with accessibility if born in a Western country compared to being born in Norway. A generally poorer patient experience of primary health care among foreign-born patients was found in previous studies [47–51]. Kjøllesdal et al. found that non-Western immigrants reported statistically significantly poorer scores on accessibility. They concluded that immigrant background is an important parameter in quality improvement work [51]. A previous international study involving 11 different countries, including Norway, has shown that patients not born in the country of residence are more likely to experience multiple barriers to accessibility to primary health care [14]. Similarly, our study shows that patients born in countries other than Norway – especially those from a non-Western country – had a negative experience related to accessibility for urgent appointments.

The GP being a specialist in general medicine was shown to have a positive association on patients’ experiences with most accessibility items. This result may be explained by the fact that a GP who is a specialist in general medicine is more experienced, which in turn can affect the patient’s experience of accessibility. These and other GP characteristics related to a negative association on the composite accessibility score in this study (female GP, on fixed salary, list length are not shown explored in other similar studies. The explorative approached we had in current study including all available GP and GP practice variables makes the study of added value in this research area. Several GP practice characteristics, such as size, composition, and functioning, are reported to represent potential levers for improving patient-reported experience in the primary healthcare setting. Our results are coherent with this study, showing that smaller practices with small number of GPs are associated with better experience of accessibility [15]. Fixed salary is more common among GPs who are younger, employed in the least central and populous municipalities, and have shorter GP lists, compared to the national average. In addition, it is more common for GPs who have fixed salaries to change work contracts, showing that these GPs offer less continuity which may also explain the negative experiences is associated to this predictor variable [52].

### Study limitations

In this study we focused on patient-experienced accessibility, not the broader concept of healthcare access. The latter is a broad and complex concept defined as “the opportunity to reach and obtain appropriate health care services in situations of perceived need for care” [7], which results from the interface between several demand-side and supply side characteristics [7]. Further research should assess broader dimensions of access, e.g., supply-side characteristics related to approachability and health care needs in vulnerable or low health literate populations. Furthermore, we only included indicators for some parts of accessibility dimensions [7], which could be broadened in future research. The regression models only explained a modest part of the variation in patient-experienced accessibility, as mesured in our study, indicating that we might lack other predictors. Further research should assess even more predictors by including more background factors about patients (e.g., income, employment status), GPs/GP pratice (e.g., staffing composition or levels) and community-level predictors like urbanity and affluence. Income at the patient-level has been shown to be associated with access to care, which we did not include in the survey [14]. We also lacked regional predictor variables like area deprivation and urbanity, although a previous study showed small effects from such factors [13].

According to Levesque`s framework [7] we addressed two of the five supply-side dimensions, but not approachability, acceptability and affordability. Some of these are challenging to measure such as approachability (the extent to which healthcare services can be identified and reached by the population), and others such as affordability are less relevant in Norway where the system is largely funded through taxes and public funds and offers many services for free or at a low cost to residents [53]. Including even more patient-level predictor variables (e.g. employment status, income, geographical distance to the GP practice/commute time) that reflect demand-side factors from Levesque`s framework such as patients’ ability to seek healthcare services when needed, could have given us more insight. However, the free text comments from our study addressed other parts of Levesque’s framework related to the demand-side factors such as the ability to reach (long travel routs to the GP) and the ability to engage (GPs professionalism, communication with the GP and not feeling safe due to absence of their own GP/without continuity). The free text comments were also related to the supply-side dimensions such as availability and accommodation, appropriateness, and acceptability (waiting time, referral, consultation time, getting though by telephone, digital communication)[7].

The strengths of the study include the use of data from a large, nationally representative survey, using a questionnaire that has been developed and tested according to established procedures. The response rate was 42%, opening up the possibility for non-response bias. However, previous research on non-response bias in patient experience surveys has indicated only modest bias related to non-response [54–58]. Moreover, a meta-analysis of the literature on survey methodology found that response rates were only a weak predictor of non-response bias in studies employing similar methodology [59].

The dependent variables were all self-reported, and the actual waiting times are unknown. The response categories for the region of birth were very broad, including many countries that probably vary in a range of measures, including the length of stay in Norway, which is unknown.

### Implication for clinical practice

This study and its results add to the knowledge of factors associated with positive patient experience of accessibility – especially continuity with their GP (including seeing their own GP and longer time on GP’s list). Further, positive patient experience is associated with patients having a GP who is a specialist in general medicine, has more years in the same contract, has shorter list length, and does not have a fixed-salary status, in addition to GP practices with lower number of GPs at the GPs practice. These results should be used to inform efforts to better organize and improve future GP practice.

## Conclusions

Key factors that are positively associated with patient-reported accessibility include patient-GP continuity including seeing own GP and more years on the GP list, better self-reported health, and shorter time since last GP contact. At the GP and GP practice- level, statistically significant predictors that are positively associated with accessibility include specialization in general medicine, while predictors negatively associated with accessibility, among others, include a higher number of GPs at the GP practice, and GPs who have a fixed-salary status. The results from the analysis of free-text comments from patients with poor accessibility scores confirmed accessibility challenges for this group, detailing and contextualizing the quantitative scores and providing additional accessibility topics and possible improvement initiatives. Based on the results from this study, both accessibility and continuity of care should be prioritized and included in future improvements of GP practice.

### Supplementary Information

Supplementary Material 1.

Supplementary Material 2.

## Data Availability

The datasets generated during and/or analysed during the current study are available from the corresponding author on reasonable request.

## References

[1] Helse- og Omsorgsdepartementet. Handlingsplan for allmennlegetjenesten - Attraktiv, kvalitetssikker og teambasert 2020–2024. Oslo 2020.

[2] Helsetilsynet. Presset fastlegesituasjon har konsekvenser for pasientene – gjennomgang av tilsynserfaringer. Rapport fra Helsetilsynet. Overstretched general practitioner situation has consequences for patients – review of supervisory experiences. Report by the Norwegian Board of Health Supervision. 3/2022,. 2022.

[3] Royal College of General Practitioners. Fit for the future. A vision for general practice. 2019. https://www.rcgp.org.uk/getmedia/1aeea016-9167-4765-9093-54a8ee8ae188/RCGP-Fit-for-the-Future-A-New-plan-for-General-Practice.pdf. Accessed 7 Jan 2024.

[4] Skudal KE SI, Bjertnæs ØA Lindahl AK, Nylenna M. Commonwealth Funds undersøkelse av helsetjenestesystemet i elleve land: Norske resultater i 2016 og utvikling over tid. Commonwealth Fund’s population survey in 11 countries: Norwegian results in 2016 and changes over time. OsloFolkehelseinstituttet; 2016.

[5] Waller A, Carey M, Mazza D, Yoong S, Grady A, Sanson-Fisher R. Patient-reported areas for quality improvement in general practice: a cross-sectional survey. Br J Gen Pract. 2015;65(634):e312–8. 25918336 10.3399/bjgp15X684841 PMC4408502

[6] Forskrift om fastlegeordning i kommunene. Regulation on the General Practitioner Scheme in Municipalities. Norway; 2012. FOR-2012-08-29-842.0.

[7] Levesque J-F, Harris MF, Russell G. Patient-centred access to health care: conceptualising access at the interface of health systems and populations. International Journal for Equity in Health. 2013;12(1):18. 23496984 10.1186/1475-9276-12-18 PMC3610159

[8] Peters DH, Garg A, Bloom G, Walker DG, Brieger WR, Rahman MH. Poverty and access to health care in developing countries. Ann N Y Acad Sci. 2008;1136:161–71. 17954679 10.1196/annals.1425.011

[9] Shengelia B, Tandon A, Adams OB, Murray CJ. Access, utilization, quality, and effective coverage: an integrated conceptual framework and measurement strategy. Soc Sci Med. 2005;61(1):97–109. 15847965 10.1016/j.socscimed.2004.11.055

[10] Penchansky R, Thomas JW. The concept of access: definition and relationship to consumer satisfaction. Med Care. 1981;19(2):127–40. 7206846 10.1097/00005650-198102000-00001

[11] Tolvanen E, Koskela TH, Mattila KJ, Kosunen E. Analysis of factors associated with waiting times for GP appointments in Finnish health centres: a QUALICOPC study. BMC Res Notes. 2018;11(1):220. 29615135 10.1186/s13104-018-3316-7 PMC5883288

[12] Premji K, Ryan BL, Hogg WE, Wodchis WP. Patients’ perceptions of access to primary care. Analysis of the QUALICOPC Patient Experiences Survey. 2018;64(3):212–20. PMC5851400 29540392

[13] Kontopantelis E, Roland M, Reeves D. Patient experience of access to primary care: identification of predictors in a national patient survey. BMC Fam Pract. 2010;11(1):61. 20799981 10.1186/1471-2296-11-61 PMC2936332

[14] Corscadden L, Levesque JF, Lewis V, Strumpf E, Breton M, Russell G. Factors associated with multiple barriers to access to primary care: an international analysis. International Journal for Equity in Health. 2018;17(1):28. 29458379 10.1186/s12939-018-0740-1 PMC5819269

[15] Cohidon C, Wild P, Senn N. Patient experience in primary care: association with patient, physician and practice characteristics in a fee-for-service system. Swiss Med Wkly. 2018;148: w14601. 29611865 10.4414/smw.2018.14601

[16] Lionis C, Papadakis S, Tatsi C, Bertsias A, Duijker G, Mekouris PB, et al. Informing primary care reform in Greece: patient expectations and experiences (the QUALICOPC study). BMC Health Serv Res. 2017;17(1):255. 28381224 10.1186/s12913-017-2189-0 PMC5382510

[17] Helsedirektoratet. Fastlegestatistikk Utviklingstrekk og endringer i fastlegeordningen: Helsedirektoratet; 2020. Available from: https://www.helsedirektoratet.no/statistikk/fastlegestatistikk. Cited 2022.

[18] Pedersen K, Godager G, Rognlien HD, Tyrihjell JB, Værnø SG, Iversen T, Holte J, Abelsen B, Pahle A, Augestad L, Sæther EM. Evaluering av handlingsplan for allmennlegetjenesten 2020–2024: Evalueringsrapport I. 2022.

[19] Norman RM, Bjertnæs, Ø. A., Danielsen, K, Holmboe, O,. Pasienterfaringer med fastlegen og fastlegekontoret i 2021/2022. [Patient experience with the general practitioner and the general practitioner office in 2021/2022]. PasOpp-rapport 2022:566. Oslo: Folkehelseinstituttet; 2022.

[20] Holmboe O, Iversen HH, Danielsen K, Bjertnaes O. The Norwegian patient experiences with GP questionnaire (PEQ-GP): reliability and construct validity following a national survey. BMJ Open. 2017;7(9): e016644. 28971964 10.1136/bmjopen-2017-016644 PMC5640105

[21] Iversen HH BØ, Holmboe O,. Pasienterfaringer med fastlegen og fastlegekontoret i 2018/19. [Patient experience with the general practitioner and the general practitioner office in 2018/19 PasOpprapport 2019:1. Oslo: Folkehelseinstituttet; 2019.

[22] Paré-Plante A-A, Boivin A, Berbiche D, Breton M, Guay M. Primary health care organizational characteristics associated with better accessibility: data from the QUALICO-PC survey in Quebec. BMC Fam Pract. 2018;19(1):188. 30509205 10.1186/s12875-018-0871-x PMC6276215

[23] Peugh JL. A practical guide to multilevel modeling. J Sch Psychol. 2010;48(1):85–112. 20006989 10.1016/j.jsp.2009.09.002

[24] Kvale S, Brinkmann S. Interviews: Learning the craft of qualitative research interviewing: sage; 2009.

[25] Fan VS, Burman M, McDonell MB, Fihn SD. Continuity of care and other determinants of patient satisfaction with primary care. J Gen Intern Med. 2005;20(3):226–33. 15836525 10.1111/j.1525-1497.2005.40135.x PMC1490082

[26] Hjortdahl P, Laerum E. Continuity of care in general practice: effect on patient satisfaction. BMJ. 1992;304(6837):1287–90. 1606434 10.1136/bmj.304.6837.1287 PMC1881840

[27] Cook LL, Golonka RP, Cook CM, Walker RL, Faris P, Spenceley S, et al. Association between continuity and access in primary care: a retrospective cohort study. CMAJ Open. 2020;8(4):E722–30. 33199505 10.9778/cmajo.20200014 PMC7676991

[28] Stephen W, Leslie B, Esther G, Susan H, Tim H, Lynda W, et al. Patient satisfaction with access and continuity of care in a multidisciplinary academic family medicine clinic. Can Fam Physician. 2014;60(4): e230. 24733343 PMC4046539

[29] Bower P, Roland M, Campbell J, Mead N. Setting standards based on patients’ views on access and continuity: secondary analysis of data from the general practice assessment survey. BMJ. 2003;326(7383):258. 12560279 10.1136/bmj.326.7383.258 PMC140766

[30] Eide TB, Straand J, Braend AM. Good communication was valued as more important than accessibility according to 707 Nordic primary care patients: a report from the QUALICOPC study. Scand J Prim Health Care. 2021;39(3):296–304. 34041993 10.1080/02813432.2021.1928837 PMC8475124

[31] Oliver D, Deal K, Howard M, Qian H, Agarwal G, Guenter D. Patient trade-offs between continuity and access in primary care interprofessional teaching clinics in Canada: a cross-sectional survey using discrete choice experiment. BMJ Open. 2019;9(3): e023578. 30904840 10.1136/bmjopen-2018-023578 PMC6475162

[32] Rubin G, Bate A, George A, Shackley P, Hall N. Preferences for access to the GP: a discrete choice experiment. Br J Gen Pract. 2006;56(531):743–8. 17007703 PMC1920713

[33] Barker I, Steventon A, Deeny SR. Association between continuity of care in general practice and hospital admissions for ambulatory care sensitive conditions: cross sectional study of routinely collected, person level data. BMJ. 2017;356: j84. 28148478 10.1136/bmj.j84

[34] Freeman F, Hughes J, editors. Continuity of care and the patient experience. 2010.

[35] Hill AP, Freeman GK. Promoting continuity of care in general practice. London: Royal College of General Practitioners; 2011.

[36] Pereira Gray D, Sidaway-Lee K, White E, Thorne A, Evans P. Improving continuity: THE clinical challenge. InnovAiT. 2016;9(10):635–45. 10.1177/1755738016654504

[37] Baker R, Freeman GK, Haggerty JL, Bankart MJ, Nockels KH. Primary medical care continuity and patient mortality: a systematic review. Br J Gen Pract. 2020;70(698):e600–11. 32784220 10.3399/bjgp20X712289 PMC7425204

[38] Wilson T, Buck D, Ham C. Rising to the challenge: will the NHS support people with long term conditions? BMJ. 2005;330(7492):657–61. 15775000 10.1136/bmj.330.7492.657 PMC554919

[39] Shi L, Starfield B. The effect of primary care physician supply and income inequality on mortality among blacks and whites in US metropolitan areas. Am J Public Health. 2001;91(8):1246–50. 11499112 10.2105/AJPH.91.8.1246 PMC1446754

[40] Shi L, Starfield B, Politzer R, Regan J. Primary care, self-rated health, and reductions in social disparities in health. Health Serv Res. 2002;37(3):529–50. 12132594 10.1111/1475-6773.t01-1-00036 PMC1434650

[41] Feng Y, Gravelle H. Patient Self-Reported Health, Clinical Quality, and Patient Satisfaction in English Primary Care: Practice-Level Longitudinal Observational Study. Value in Health. 2021;24(11):1660–6. 34711367 10.1016/j.jval.2021.05.019

[42] Guthrie B, Wyke S. Personal continuity and access in UK general practice: a qualitative study of general practitioners’ and patients’ perceptions of when and how they matter. BMC Fam Pract. 2006;7(1):11. 16504130 10.1186/1471-2296-7-11 PMC1413534

[43] Thompson AE, Anisimowicz Y, Miedema B, Hogg W, Wodchis WP, Aubrey-Bassler K. The influence of gender and other patient characteristics on health care-seeking behaviour: a QUALICOPC study. BMC Fam Pract. 2016;17(1):38. 27036116 10.1186/s12875-016-0440-0 PMC4815064

[44] Deveugele M, Derese A, van den Brink-Muinen A, Bensing J, De Maeseneer J. Consultation length in general practice: cross sectional study in six European countries. BMJ. 2002;325(7362):472. 12202329 10.1136/bmj.325.7362.472 PMC119444

[45] Nabalamba A, Millar WJ. Going to the doctor. Health Rep. 2007;18(1):23–35. 17441441

[46] Weisman CS, Rich DE, Rogers J, Crawford KG, Grayson CE, Henderson JT. Gender and Patient Satisfaction with Primary Care: Tuning in to Women in Quality Measurement. J Womens Health Gend Based Med. 2000;9(6):657–65. 10957754 10.1089/15246090050118189

[47] Magadi JP, Magadi MA. Ethnic inequalities in patient satisfaction with primary health care in England: Evidence from recent General Practitioner Patient Surveys (GPPS). PLoS ONE. 2022;17(12): e0270775. 36542601 10.1371/journal.pone.0270775 PMC9770381

[48] Pinder RJ, Ferguson J, Møller H. Minority ethnicity patient satisfaction and experience: results of the National Cancer Patient Experience Survey in England. BMJ Open. 2016;6(6): e011938. 27354083 10.1136/bmjopen-2016-011938 PMC4932347

[49] Lyratzopoulos G, Elliott M, Barbiere JM, Henderson A, Staetsky L, Paddison C, et al. Understanding ethnic and other socio-demographic differences in patient experience of primary care: evidence from the English General Practice Patient Survey. BMJ Qual Saf. 2012;21(1):21–9. 21900695 10.1136/bmjqs-2011-000088 PMC3240774

[50] Lien E, Nafstad P, Rosvold EO. Non-western immigrants’ satisfaction with the general practitioners’ services in Oslo, Norway. International Journal for Equity in Health. 2008;7(1):7. 18304309 10.1186/1475-9276-7-7 PMC2292726

[51] Kjøllesdal M, Indseth T, Iversen HH, Bjertnaes O. Patient experiences with general practice in Norway: a comparison of immigrant groups and the majority population following a national survey. BMC Health Serv Res. 2020;20(1):1106. 33256725 10.1186/s12913-020-05963-3 PMC7708102

[52] Skyrud KD RT. Utvikling i fastlegenes arbeidsmengde og situasjon over tid. [Development in general practitioners’ workload and situation over time] Rapport 2023. . Oslo: Folkehelseinstituttet; 2023.

[53] Healthcare in Norway. The Norwegian Directorate of Health; Oslo 2023. Available from: https://www.helsenorge.no/en/healthcare/.

[54] Garratt AM, Bjertnaes OA, Holmboe O, Hanssen-Bauer K. Parent experiences questionnaire for outpatient child and adolescent mental health services (PEQ-CAMHS Outpatients): reliability and validity following a national survey. Child Adolesc Psychiatry Ment Health. 2011;5(1):18. 21600010 10.1186/1753-2000-5-18 PMC3120777

[55] Bjertnaes ØA, Garratt A, Johannessen JO. Data collection methods and results in user surveys in mental health care. Tidsskr Nor Laegeforen. 2006;126(11):1481–3. 16732343

[56] Bjertnaes OA, Garratt A, Botten G. Nonresponse bias and cost-effectiveness in a Norwegian survey of family physicians. Eval Health Prof. 2008;31(1):65–80. 18174607 10.1177/0163278707311874

[57] Bjertnaes OA, Garratt A, Iversen H, Ruud T. The association between GP and patient ratings of quality of care at outpatient clinics. Fam Pract. 2009;26(5):384–90. 19584122 10.1093/fampra/cmp043 PMC2743734

[58] Guldvog B, Hofoss D, Pettersen KI, Ebbesen J, Rønning OM. PS-RESKVA (Patient Satisfaction, Results and Quality)–patient satisfaction in hospitals. Tidsskr Nor Laegeforen. 1998;118(3):386–91. 9499727

[59] Groves RM, Peytcheva E. The Impact of Nonresponse Rates on Nonresponse Bias: A Meta-Analysis. Public Opin Q. 2008;72(2):167–89. 10.1093/poq/nfn011

