# Supplementary material for: Factors associated with patients’ experience of accessibility to general practice: results from a national survey in Norway
Source: BMC Health Serv Res. 2024 Aug 30;24:1008. doi: 10.1186/s12913-024-11460-8 (PMC11365193; doi:10.1186/s12913-024-11460-8)
Supplement: Supplementary file 1 — Supplementary Material 1. [file 12913_2024_11460_MOESM1_ESM.docx]

| **Dependent variables** | **Urgent appointment with**  **your GP within same or next day (n= 5728)** | | | **Waiting time for an urgent appointment acceptable**  **(n= 5726)** | | | **Usual waiting time for an appointment within 7 days**  **(n= 6469)** | | | **Waiting time for a regular appointment acceptable**  **(n= 6443)** | | | **Enough time with the GP**  **(n= 6864)** | | | **Waiting time (none or small extent) in the**  **waiting room (n= 6842)** | | | **Difficulty (none or small extent) in getting in touch with your GPs office by telephone (n= 6706)** | | |
| --- | --- | --- | --- | --- | --- | --- | --- | --- | --- | --- | --- | --- | --- | --- | --- | --- | --- | --- | --- | --- | --- |
| **Predictor variables** | **OR** | **95 % CI for OR (LL)** | **95 % CI for OR (UL)** | **OR** | **95 % CI for OR (LL)** | **95 % CI for OR (UL)** | **OR** | **95 % CI for OR (LL)** | **95 % CI for OR (UL)** | **OR** | **95 % CI for OR (LL)** | **95 % CI for OR (UL)** | **OR** | **95 % CI for OR (LL)** | **95 % CI for OR (UL)** | **OR** | **95 % CI for OR (lower)** | **95 % CI for OR (upper)** | **OR** | **95 % CI for OR (LL)** | **95 % CI for OR (UL)** |
| **Sex**  Male (RG) |  |  |  |  |  |  |  |  |  |  |  |  |  |  |  |  |  |  |  |  |  |
| Female | **1.184**** | **1.064** | **1.319** | **1.118**** | **1.000** | **1.249** | **0.616***** | **0.558** | **0.681** | **0.846***** | **0.766** | **0.934** | **0.884**** | **0.793** | **0.986** | **0.731***** | **0.662** | **0.806** | **0.750***** | **0.676** | **0.832** |
| **Age** | **0.996**** | **0.993** | **0.999** | **0.992***** | **0.988** | **0.995** | 1.002 | 0.999 | 1.006 | **1.003**** | **1.000** | **1.006** | **1.005**** | **1.001** | **1.008** | **1.019***** | **1.016** | **1.022** | **1.010***** | **1.007** | **1.013** |
| **Education**  (Primary school RG) |  |  |  |  |  |  |  |  |  |  |  |  |  |  |  |  |  |  |  |  |  |
| High school | **1.202**** | **1.020** | **1.416** | 1.156 | 0.978 | 1.367 | 0.967 | 0.826 | 1.132 | 0.995 | 0.852 | 1.163 | 0.928 | 0.784 | 1.099 | 0.961 | 0.825 | 1,120 | 0.889 | 0.756 | 1,046 |
| University (1-4 years) | **1.191**** | **1.003** | **1.414** | **1.317**** | **1.104** | **1.570** | **0.833**** | **0.708** | **0.980** | 1.129 | 0.961 | 1.327 | 0.922 | 0.774 | 1.099 | 0.877 | 0.748 | 1,029 | 0.919 | 0.776 | 1.088 |
| University (more than 4 years) | 1.145 | 0.951 | 1.378 | **1.425***** | **1.176** | **1.727** | **0.677***** | **0.569** | **0.806** | 1.102 | 0.927 | 1.310 | 0.907 | 0.751 | 1.096 | **0.785**** | **0.660** | **0.933** | 0.865 | 0.722 | 1.037 |
| **Number of chronic conditions**  No (RG) |  |  |  |  |  |  |  |  |  |  |  |  |  |  |  |  |  |  |  |  |  |
| 1 | 0.946 | 0.823 | 1.088 | **1.162**** | **1.006** | **1.344** | **0.822**** | **0.722** | **0.936** | 1.037 | 0.913 | 1.179 | 1.006 | 0.873 | 1.158 | 0.949 | 0.835 | 1.078 | 0.901 | 0.788 | 1.030 |
| 2 | 0.918 | 0.777 | 1.084 | 1.166 | 0.982 | 1.385 | **0.768***** | **0.659** | **0.895** | 1.031 | 0.885 | 1.200 | 1.076 | 0.910 | 1.273 | 0.873 | 0.750 | 1.017 | 0.978 | 0.834 | 1.148 |
| More than two | 1.041 | 0.858 | 1.261 | 1.175 | 0.965 | 1.429 | **0.707***** | **0.591** | **0.846** | 1.145 | 0.957 | 1.369 | 1.020 | 0.842 | 1.237 | 0.917 | 0.768 | 1.094 | 0.909 | 0.756 | 1.093 |
| **Country of Birth**  Norway (RG) |  |  |  |  |  |  |  |  |  |  |  |  |  |  |  |  |  |  |  |  |  |
| Western countries | 0.905 | 0.739 | 1.108 | **0.733**** | **0.596** | **0.901** | **1.948***** | **1.590** | **2.387** | 1.045 | 0.864 | 1.264 | **1.237**** | **1.002** | **1.528** | **1.287**** | **1.065** | **1.555** | **1.412***** | **1.147** | **1.738** |
| Non- Western countries | 0.820 | 0.658 | 1.022 | **0.449***** | **0.360** | **0.559** | **2.154***** | **1.705** | **2.720** | **0.643***** | **0.515** | **0.803** | 0.914 | 0.729 | 1.146 | **1.365**** | **1.098** | **1.697** | 1.149 | 0.920 | 1.436 |
| **Self-reported physical heath** | 1.010 | 0.937 | 1.090 | **1.164***** | **1.077** | **1.258** | 1.029 | 0.959 | 1.104 | **1.202***** | **1.119** | **1.290** | **1.226***** | **1.137** | **1.322** | **1.124***** | **1.047** | **1.206** | 1.066 | 0.991 | 1.146 |
| **Self-reported mental health** | **1.106**** | **1.033** | **1.185** | **1.162***** | **1.083** | **1.247** | 1.022 | 0.959 | 1.090 | **1.150***** | **1.078** | **1.226** | **1.362***** | **1.272** | **1.458** | **1.178***** | **1.103** | **1.258** | **1.161***** | **1.087** | **1.240** |
| **Years on GP list** | **1.025***** | **1.017** | **1.033** | **1.028***** | **1.020** | **1.036** | 1.003 | 0.996 | 1.010 | **1.023***** | **1.016** | **1.030** | **1.022***** | **1.014** | **1.031** | 0.997 | 0.990 | 1.004 | **1.012***** | **1.005** | **1.020** |
| **Time since last contact with the GP**  Less than a month (RG) |  |  |  |  |  |  |  |  |  |  |  |  |  |  |  |  |  |  |  |  |  |
| 1-3 months | 0.898 | 0.792 | 1.018 | 0.934 | 0.821 | 1.064 | 1.080 | 0.962 | 1.213 | 0.938 | 0.836 | 1.052 | 0.940 | 0.828 | 1.067 | 0.937 | 0.836 | 1.052 | 0.905 | 0.803 | 1.021 |
| 4-6 months | 0.876 | 0.747 | 1.027 | 0.927 | 0.786 | 1.094 | 0.994 | 0.858 | 1.153 | 1.007 | 0.870 | 1.166 | **0.833**** | **0.710** | **0.979** | 0.868 | 0.750 | 1.006 | **0.829**** | **0.712** | **0.966** |
| 7-12 months | **0.552**** | **0.449** | **0.679** | **0.729**** | **0.589** | **0.901** | 0.970 | 0.804 | 1.169 | 0.853 | 0.708 | 1.027 | **0.760**** | **0.623** | **0.928** | 0.948 | 0.790 | 1.139 | **0.837** | **0.691** | **1.015** |
| More than 12 months | 0.791 | 0.576 | 1.086 | 0.710 | 0.513 | 0.982 | 0.914 | 0.669 | 1.250 | 0.813 | 0.591 | 1.119 | **0.608***** | **0.448** | **0.824** | **0.649**** | **0.471** | **0.893** | **0.738**** | **0.545** | **1.000** |
| **Do you usually meet your own GP?** | **2.010***** | **1.719** | **2.349** | **2.596***** | **2.215** | **3.042** | **1.811***** | **1.557** | **2.106** | **2.536***** | **2.153** | **2.988** | **3.210***** | **2.764** | **3.728** | **1.858***** | **1.573** | **2.193** | **2.099***** | **1.808** | **2.436** |

**OR=Odds ratio, CI = confidence interval; LL = lower limit; UL = upper p<0.05*, p<0.01**, p <0.001***, in bold.**

Supplemental table 1. Multivariate logistic regression from the seven accessibility items as dependent variables and patient characteristics as predictors variables.
