# Supplementary material for: Factors associated with patients’ experience of accessibility to general practice: results from a national survey in Norway
Source: BMC Health Serv Res. 2024 Aug 30;24:1008. doi: 10.1186/s12913-024-11460-8 (PMC11365193; doi:10.1186/s12913-024-11460-8)
Supplement: Supplementary file 2 — Supplementary Material 2. [file 12913_2024_11460_MOESM2_ESM.docx]

| **Dependent variables** | **Urgent appointment with**  **your GP within same or next day (n= 5728)** | | | **Waiting time for an urgent appointment acceptable**  **(n= 5726)** | | | **Usual waiting time for an appointment within 7 days**  **(n= 6469)** | | | **Waiting time for a regular appointment acceptable**  **(n= 6443)** | | | **Enough time with the GP**  **(n= 6864)** | | | **Waiting time (none or small extent) in the**  **waiting room (n= 6842)** | | | **Difficulty (none or small extent) in getting in touch with your GPs office by telephone (n= 6706)** | | |
| --- | --- | --- | --- | --- | --- | --- | --- | --- | --- | --- | --- | --- | --- | --- | --- | --- | --- | --- | --- | --- | --- |
| **Predictor variables** | **OR** | **95 % CI for OR LL** | **95 % CI for OR UL** | **OR** | **95 % CI for OR LL** | **95 % CI for OR UL** | **OR** | **95 % CI for OR LL** | **95 % CI for OR UL** | **OR** | **95 % CI for OR LL** | **95 % CI for OR UL** | **OR** | **95 % CI for OR LL** | **95 % CI for OR UL** | **OR** | **95 % CI for OR LL** | **95 % CI for OR UL** | **OR** | **95 % CI for OR LL** | **95 % CI for OR UL** |
| **GPs age** | 0.996 | 0.988 | 1.004 | 0.998 | 0.990 | 1.005 | 1.000 | 0.993 | 1.007 | 1.001 | 0.994 | 1.008 | 0.999 | 0.992 | 1.007 | 0.996 | 0.989 | 1.003 | 0.994 | 0.987 | 1.001 |
| **GPs sex**  Male (RG) |  |  |  |  |  |  |  |  |  |  |  |  |  |  |  |  |  |  |  |  |  |
| Female | 1.012 | 0.910 | 1.126 | 1.008 | 0.904 | 1.124 | **0.697***** | **0.631** | **0.770** | **0.807***** | **0.732** | **0.891** | 0.942 | 0.848 | 1.046 | **0.802***** | **0.728** | **0.884** | **0.879**** | **0.794** | **0.974** |
| **Years in the same employment contract** | **1.014**** | **1.002** | **1.027** | **1.020**** | **1.007** | **1.032** | 0.997 | 0.986 | 1.008 | **1.011**** | **1.000** | **1.022** | **1.016**** | **1.004** | **1.028** | 0.998 | 0.987 | 1.009 | **1.014**** | **1.003** | **1.026** |
| **List length**  0-499 (RG) |  |  |  |  |  |  |  |  |  |  |  |  |  |  |  |  |  |  |  |  |  |
| 500-999 | 0.970 | 0.752 | 1.251 | 1.202 | 0.931 | 1.552 | **0.756**** | **0.589** | **0.969** | 0.870 | 0.683 | 1.108 | 1.034 | 0.808 | 1.323 | 0.832 | 0.658 | 1.052 | 0.826 | 0.641 | 1.065 |
| 1000-1499 | 0.940 | 0.717 | 1.232 | 1.039 | 0.791 | 1.364 | 0.809 | 0.621 | 1.054 | 0.809 | 0.626 | 1.045 | 0.838 | 0.644 | 1.091 | 0.789 | 0.615 | 1.014 | **0.655**** | **0.500** | **0.858** |
| Over 1500 | 0.962 | 0.702 | 1.318 | 1.040 | 0.757 | 1.429 | 0.961 | 0.707 | 1.307 | 0.800 | 0.596 | 1.074 | **0.697**** | **0.514** | **0.944** | 0.794 | 0.596 | 1.059 | **0.573***** | **0.421** | **0.781** |
| **Number of available spots on the GPs list**  0 (RG) |  |  |  |  |  |  |  |  |  |  |  |  |  |  |  |  |  |  |  |  |  |
| 1-10 | 0.906 | 0.801 | 1.025 | 0.962 | 0.848 | 1.091 | 1.094 | 0.977 | 1.225 | 1.048 | 0.936 | 1.172 | 0.921 | 0.816 | 1.041 | 1.057 | 0.946 | 1.181 | 0.960 | 0.854 | 1.078 |
| 11-99 | **0.730**** | **0.592** | **0.900** | **0.747**** | **0.604** | **0.924** | **1.383**** | **1.131** | **1.691** | 0.948 | 0.780 | 1.152 | **0.622***** | **0.510** | **0.759** | 0.835 | 0.688 | 1.015 | 0.829 | 0.679 | 1.013 |
| 100 and more | **0.800**** | **0.665** | **0.963** | **0.846** | **0.701** | **1.021** | **1.587***** | **1.325** | **1.900** | 0.997 | 0.840 | 1.184 | **0.676***** | **0.565** | **0.809** | 0.981 | 0.829 | 1.161 | 0.839 | 0.702 | 1.002 |
| **Number of GPs at the GP practice** | 0.976 | 0.949 | 1.004 | 0.982 | 0.954 | 1.011 | **0.905***** | **0.881** | **0.929** | **0.931***** | **0.907** | **0.956** | **0.972**** | **0.946** | **0.999** | **0.947***** | **0.923** | **0.972** | **0.913***** | **0.889** | **0.937** |
| **General medicine specialist** No (RG) |  |  |  |  |  |  |  |  |  |  |  |  |  |  |  |  |  |  |  |  |  |
| Yes | **1.348***** | **1.189** | **1.527** | **1.358***** | **1.196** | **1.542** | **1.127**** | **1.001** | **1.269** | **1.345***** | **1.198** | **1.511** | **1.215**** | **1.076** | **1.371** | **1.249***** | **1.113** | **1.401** | **1.419***** | **1.260** | **1.599** |
| **Fixed salary**  No (RG) |  |  |  |  |  |  |  |  |  |  |  |  |  |  |  |  |  |  |  |  |  |
| Yes | **0.726***** | **0.609** | **0.866** | **0.736***** | **0.616** | **0.879** | **0.678***** | **0.574** | **0.801** | **0.685***** | **0.579** | **0.809** | **0.842**** | **0.710** | **1.000** | 1.066 | 0.907 | 1.254 | 0.930 | 0.784 | 1.102 |
| **Group GP practice**  No (RG) |  |  |  |  |  |  |  |  |  |  |  |  |  |  |  |  |  |  |  |  |  |
| Yes | 1.040 | 0.864 | 1.252 | 0.959 | 0.793 | 1.160 | **0.633***** | **0.523** | **0.766** | **0.805**** | **0.677** | **0.958** | 0.880 | 0.730 | 1.061 | **0.792**** | **0.669** | **0.937** | 0.855 | 0.711 | 1.029 |
| **Joint GP list**  No (RG) |  |  |  |  |  |  |  |  |  |  |  |  |  |  |  |  |  |  |  |  |  |
| Yes | 1.046 | 0.797 | 1.373 | 1.054 | 0.800 | 1.389 | 0.969 | 0.753 | 1.248 | 1.055 | 0.819 | 1.359 | 1.063 | 0.815 | 1.387 | **1.275**** | **1.000** | **1.626** | **1.460**** | **1.107** | **1.925** |

**OR=Odds ratio, CI = confidence interval; LL = lower limit; UL = upper p<0.05*, p<0.01**, p <0.001***, in bold.**

Supplemental table 2. Multivariate logistic regression from the seven accessibility items as dependent variables and GP and GP practice characteristics as predictors variables.
